# Solving the 'Goldilocks problem' in dementia clinical trials with multimodal AI

**DOI:** 10.1016/j.tjpad.2025.100397

**Published:** 2025-12-01

**Authors:** Andrew E. Welchman, Zoe Kourtzi

**Affiliations:** a Prodromic Ltd, Milton Hall, Ely Road, Milton, Cambridge CB24 6WZ, UK; b Department of Psychology, University of Cambridge, Cambridge CB2 3 EB, UK

**Keywords:** Multimodal AI, Clinical trials, Imaging, Cognition, Alzheimer's disease, Early prediction

## Abstract

The development of effective therapeutics for Alzheimer’s Disease and related dementias (ADRD) has been hindered by patient heterogeneity and the limitations of current diagnostic tools. New treatments have no chance of working if given to patients who cannot benefit from them. This perspective explores how advances in Artificial Intelligence (AI), particularly multimodal machine learning, can solve the ‘Goldilocks problem’ of identifying patients for inclusion in clinical trials and support precision treatment in real-world healthcare settings. We examine the challenges of patient stratification, grounded by a conceptual framework of identifying each person’s stage and subtype of dementia. We review data from several clinical trials of Alzheimer’s disease therapeutics, to explore how AI-guided patient stratification can improve trial outcomes, reduce costs and improve recruitment. Further, we discuss the integration of AI into clinical workflows, the importance of model interpretability and generalizability, and ethical imperative to address algorithmic bias. By combining AI with scientific insight, clinical expertise, and patient experience, we argue that intelligent analytics can accelerate the discovery and delivery of new diagnostics and therapeutics, ultimately transforming dementia care and improving outcomes for patients around the globe.

## 1 Introduction

Developing new therapeutics for Alzheimer’s Disease (AD) has been hampered by patient heterogeneity and a lack of sensitive tools to precisely stratify and separate individual patients. Despite scientific progress in promising new drug candidates, clinical trials of potential disease-modifying treatments have been disproportionately unsuccessful [1].

In some cases, patients included in trials were mistakenly believed to have AD, when in fact they did not. In other cases, included patients were too far progressed to benefit from the therapeutic’s mechanism of action.

This ‘Goldilocks problem’—finding patients who are neither too early nor too late in disease progression—lies at the heart of the challenge of identifying appropriate patients for (a) inclusion in clinical trials and (b) prescription of therapeutics in real world healthcare settings. Good drugs fail if given to patients who have no possibility of benefitting from them. Ongoing efforts are seeking to use biomarkers to improve accurate identification of dementias (e.g [2,3]). Here we ask, how recent advances in Artificial Intelligence (AI) and machine learning can help. We explore how AI-guided patient stratification can accelerate the development and real-world impact of new therapeutics for AD and related dementias (ADRD). We structure this article around three themes:

(1) Improving the sensitivity of clinical trials to gain a better understanding of the efficacy of a medication for the target population.
(2) Improving the efficiency of clinical trials to accelerate therapeutic development.
(3) Improving the real-world effectiveness of a medication in clinical practice.

We start by framing the problem of patient stratification in dementias conceptually, to expose the complexity of the challenges that need to be solved, and to explore how AI can help.

### 1.1 Why dementia is a hard problem

**Fig. 1:**
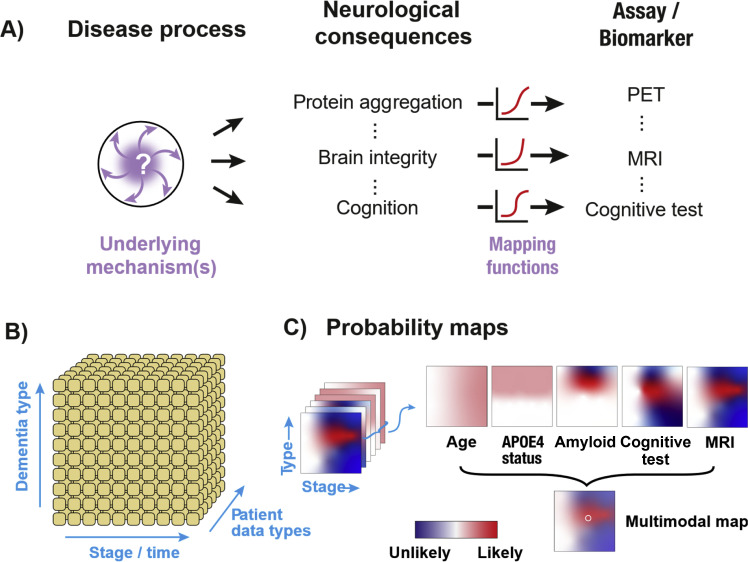
A) Illustration depicting how an underlying pathology can relate to its measurable biomarkers through a range of different ‘mapping functions’, shown by different shaped curves. B) A schematic of the conceptual space within which an individual patient can be located – a specific subtype of dementia (y-axis), at a specific stage (x-axis), where data are obtained across a range of different assays and markers (z-axis). C) Illustration of probability maps describing the relationship between information from particular data types in relation to the space of subtypes and disease stages (not empirical data). Different biomarkers provide different degrees of certainty in particular parts of the space shown by variations in the saturation of the colours. White indicates locations where the marker provides no information, saturated coloured regions represent likely (red) or unlikely (blue) disease locations for the patient. Data providing a precise signal to the patient’s dementia type and stage would be shown as a small, sharp red point surrounded by dark blue, while a blurred, desaturated region indicates uncertainty about the patient’s true location. Combining the diverse data types results in a multimodal map that accumulates all of the signals. Reading out the peak (white circle) indicates the individual’s most probable subtype and stage.

Dementia results from a cascade of processes that are not fully understood (Fig. 1a). The underlying disease process gives rise to multiple neurological consequences ranging from physical to psychological. To quantify these, the field has adopted a range of measurements (e.g., using Positron Emission Tomography (PET) scans, Magnetic Resonance Imaging (MRI), blood tests, Cerebrospinal Fluid samples, Cognitive tests) that relate (in ways not fully understood) to the underlying pathology.

Further, the ‘mapping function’—the relationship between the underlying pathology and its measurable biomarkers—is complex and non-linear. For instance, a biomarker assay may be insensitive to low concentrations of a protein, while changes in large quantities of the protein may be measurable but not functionally meaningful. Different assay domains (e.g., protein, brain structure, cognitive construct) quantify different aspects of the underlying pathology and are subject to different mapping functions. As a result, the interrelation between different assays (e.g., the correspondence between a cognitive test and measures of the accumulation of amyloid beta (β-Amyloid) in the brain) is highly complex.

To identify patients that could benefit from a potential new treatment, we are faced with the ‘inverse problem’ of using measurable information about the individual (‘patient data’ such as demographics, medical history, biomarkers) and working backwards to estimate a given patient’s disease status. As current biomarkers lack precision, this gives rise to considerable uncertainty – demonstrated by high rates of misdiagnosis at early stages of dementia [4].

The problem is particularly acute in dementia because of a range of conditions (e.g., AD, Lewy Body Dementia, Frontotemporal Dementia) may appear similar in prodromal phases, and each is likely to have subtypes (e.g., subtypes of AD [5]) and/or co-pathologies that are not yet fully understood. Moreover, because dementias involve a cascade of processes responsible for different pathologies, understanding the timing of the disease is critical to ensure that a drug’s mechanism of action (e.g. reduced β-Amyloid synthesis) is appropriate for the patient’s current main driver of pathology.

### 1.2 How to tackle the challenge

We conceptualise the core problem as pinpointing an individual’s position (Fig. 1b) in the space of dementia stages / time (*x*-axis) and subtypes (*y*-axis). To do this, we accumulate information across a range of data types (*z*-axis), each of which provides only partial and/or ambiguous signals about the patient’s disease status. We can think of the information provided by each data type as a probabilistic cue to the true position of the patient in the stage-by-subtype space, visualised as probability maps.

Different types of data provide information in different parts of the space (Fig. 1c). Age, for instance, provides a weak signal (pale pink image intensities) to stage, and does not provide information about subtype. By contrast, Apolipoprotein E allele E4 (ApoE4) status makes some subtypes of dementia more likely but provides no information about a patient’s stage. Information about Aβ burden from a PET scan (or blood test) will be weakly predictive at early disease stages, then provide information that can help pinpoint dementia type, with some indication of stage, but thereafter only provide weak information about a patient’s stage.

How should we make use of this information? The space of possible biomarkers, subtypes and stages has the potential to be overwhelming. A simple approach for human decision-making is therefore to set thresholds on specific signals, and then sequentially examine a series of markers to see if they are out of range. This is relatively easy to implement (a series of “If… then…” rules that form a decision tree), but can compromise the sensitivity and specificity of clinical decisions. In particular, single biomarkers are unlikely to capture the whole disease process (Fig. 1), so clinical outcomes rarely depend on a single measurement. We therefore need to consider interactions between different signals. Doing this with a decision tree rapidly becomes complex when there are multiple variables (e.g., age, sex, co-pathologies, amyloid, cognition) – creating dense ‘branches’ to capture all of the possible outcomes for each decision. Moreover, medical information is subject to uncertainty (i.e., it is not perfect): fluctuations in signals and measurement error mean that an erroneous decision from rigid linear cutoffs at an early binary (yes/no) decision stage could lead to a patient being fundamentally misclassified. Finally, fixed thresholds can be particularly problematic for patients whose background characteristics (e.g., ethnicity) are not well represented in the normative samples used to establish threshold values [6]. Together these limitations necessitate a more probabilistic approach that simultaneously combines information from different signals – i.e., a multimodal approach.

Machine learning (ML) methods are inherently well suited to identifying patterns in large, multi-dimensional and multi-modal datasets. They can learn optimal boundaries from data, accommodating multiple predictors in ways that are robust to the uncertainties inherent in clinical data. Given a sufficiently large sample, they will approximate the functions linking measured data and outcomes. In particular, by analysing many patient records composed of different biomarkers and clinical labels, they will learn the probabilistic (data-driven) relationships between markers and outcomes. In this way, the conceptual framework described by Fig. 1 can be made explicit by learning from clinical data. The specific approach taken depends on the data types and the availability and/or certainty of clinical labels (i.e. diagnosis). When clinical labels are available and reliable, associating data with these labels (supervised learning) provides a reliable way to derive a patient’s most likely dementia type and clinical stage. In other circumstances (e.g., lack of reliable labels), unsupervised or semi-supervised methods can facilitate the discovery of groupings in the data (‘latent classes’) that reveal new insights into a patient’s specific dementia subtype or clinical stage. This later approach has particular potential to discover new subtypes and/or distinct stages that can, in turn, be related to new biological insights from specific biomarkers (e.g. neuroinflammation, blood, proteomics markers) to improve diagnostic crieria [7].

The viability of these ML approaches has improved dramatically over the past decade thanks to the systematic accumulation of large-scale, high quality data resources. Initiatives such as the AD Neuroimaging Initiative (ADNI) [8], the US National Alzheimer’s Coordinate Center (NACC) repository [9], the AD Data Initiative (ADDI) [10], and the Dementias Platform UK (DPUK) [11] have opened up the potential to understand how multimodal markers of ADRD are expressed across large populations of individuals. In particular, carefully curated, multivariate data with clinical labels provides a rich workspace with which to understand the presentation and progression of different forms of dementias using AI methods. How can we leverage these data to improve the search for new treatments?

## 2 Improving clinical trial sensitivity

The past decade has seen remarkable progress in developing therapeutics that target biological markers associated with AD (e.g., β-Amyloid), but with disappointing results in terms of altering functional (cognitive) symptoms of the disease. Therapies designed to reduce Amyloid production through β-secretase enzyme inhibition (BACE inhibitors) showed efficacy in lowering Amyloid levels within the central nervous system (e.g., Lanabecestat [12], Verubecestat [13,14], Atabecestat [15]), but were not found to positively impact cognition. Other approaches have used Aβ immunotherapies to target the accumulation of Amyloid-beta plaques within the central nervous system. While these have proved effective in engaging their biological targets and reducing β-Amyloid (e.g., Aducanumab [16], Gantenerumab [17]), only two—Lecanemab [18] and Donanemab [19]—slowed down the rate of cognitive decline. What is responsible for this discrepancy between impact on biomarker levels of β-Amyloid and functional symptoms of the disease (cognition)?

There are several logical possibilities: (1) reductions in Aβ may have been insufficient; (2) the chosen biomarkers may be poor surrogates for the disease; (3) cognitive measures (trial endpoints) may lack sensitivity, introducing variation that masks the treatment effect; (4) differences between patients (heterogeneity) may introduce variation that makes it harder to detect true benefits from a treatment – i.e., between-patient variability masks the treatment effect. Here, we focus on how ML approaches help tackle the last of these possibilities by derisking clinical trials and enhancing their efficiency and efficacy.

**Fig. 2:**
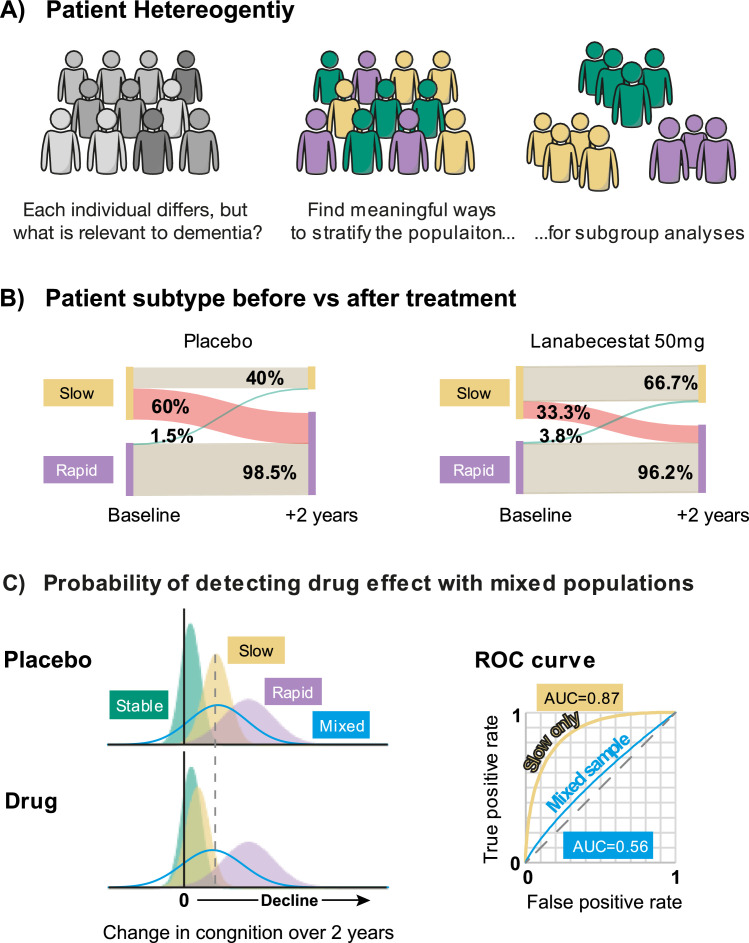
A) Illustration of the problem of patient heterogeneity. We know individuals differ, but we need to identify the axes along which to segment them into meaningful subtype groupings. B) Alluvial plots reproduced from Vaghari et al [20] illustrating transitions between ‘Slow’ and ‘Rapid’ patient classifications at the start (baseline) and end (+2 years) of the AMARANTH trial. The width of the line indicates the proportion of patients in the category. C) Modelling the effects of mixed populations on the ability to detect the effect of a drug that only benefits ‘Slow’ progressive patients. The left portion of the figure represents probability density for the change in cognition over a two year period. The right portion of the plot shows an ROC curve considering the whole sample (blue curve) or limiting it to comparing the ‘Slow’ progressive patients (yellow curve). We quantify performance in terms of the Area Under the Curve, where 0.5 represents chance and 1 perfect performance.

In a recent study, we leveraged a multimodal ML approach [20] to determine whether precision stratification can reveal a treatment effect in a trial judged to be futile. The study re-examined data from the AMARANTH phase 2/3 trial (ClinicalTrials.gov ID: NCT02245737) of the BACE1 inhibitor Lanabecestat (AZD3293, LY3314814). While the trial had shown successful lowering of β-amyloid, there was no statistically reliable slowing of cognitive decline (the study’s primary endpoint) [12]. To determine whether this was due to patient heterogeneity (Fig. 2a), we applied a previously-trained ML model [[21], [22], [23]] to patient data at baseline (i.e., before administration of the drug or placebo). The model used the standard clinical data collected in the trial (structural MRI, florbetapir PET (β-amyloid), ApoE4) to derive an individualised prediction for each patient’s future cognitive health. This AI-guided marker predicts progression from early stages of disease (Mild Cognitive Impairment and even pre-symptomatic, Cognitive Normal) to AD more precisely than standard clinical assessments [23] and biomarkers typically used in clinical trials [22]. Thereby, patients were classified as either ‘stable’ (i.e., unlikely to deteriorate) or ‘progressive’, where progression was identified as ‘slow’ or ‘rapid’.

Reanalysing the trial data based on stratified subgroups identified an effect on cognitive outcomes for ‘slowly progressive’ patients. In particular, the stratified analysis revealed a 46 % slowing of cognitive decline as measured by the Clinical Dementia Rating – Sum of Boxes scale (the original primary endpoint for the study) that was specific to patients on a slowly progressive trajectory. Importantly, the model had been trained and optimised on an entirely separate data set (research data from ADNI) with the task of predicting a patient’s future dementia trajectory. The fact that this generalised to a new context for a different task (identifying the effectiveness of a drug) suggests the ML model identifies biologically-meaningful clusters of patients. In particular, ‘slowly progressive’ patients appear to be functionally responsive to a therapeutic that reduces β-amyloid, while rapidly progressive patients do not.

How should we understand ‘slowly progressive’ and ‘rapidly progressive’ patient groups in relation to the stage-by-subtype space (Fig. 1b)? Do these statistically-inferred groups relate to biologically meaningful distinctions? It is logically possible that ‘slow’ vs. ‘rapid’ form different subtypes of Alzheimer’s disease (i.e., different positions along the *y*-axis), and/or that they represent different stages in the progression of the disease (different positions along the *x*-axis). A deeper dive into biomarkers associated with different subtypes could help determine this definitively: for instance, conducting in depth assays on a wide range of multi-omics, cognition, co-pathologies, neuroinflammation, neuroprotective markers to determine whether biologically distinct mechanisms underlie AI-identified subtypes. Understanding interactions between these factors (as extracted and learned by the AI models) will allow us to determine precisely different subtypes (and potentially discover new ones) that may have different underlying mechanisms. Nevertheless, longitudinal data from a real-world memory clinic [23] and monitoring progression across the duration of the AMARANTH study [20] suggest that slow vs. rapid progressive groups relate to different disease stages. In particular, reclassifying trial participants at the end of the study indicated different probabilities of transitioning from ‘slow’ to ‘rapid’ (Fig. 2b). For patients in the placebo group, sixty percent of the patients identified as ‘slow’ at baseline had transitioned to ‘rapid’ at the conclusion of the study – i.e., suggestive of a change of dementia stage rather than subtype. For those given the highest dose of Lanabecestat, only a third of patients transitioned from ‘slow’ to ‘rapid’ at the conclusion of the study, suggesting that lowering β-Amyloid slows down the progressive nature of AD. This analysis provides an initial indication that prognostic scores derived from multimodal AI models could serve as clinical trial endpoints, delivering multimodal markers that are more sensitive for testing treatment effects than single modality markers alone (e.g. cognitive tests).

Further, this use of longitudinal datasets points to a broader opportunity to develop machine learning approaches for precise patient stratification; that is, modelling longitudinal data to predict individualized trajectories, rather than relying on risk factors at population level or progression rates derived from previous studies. The stratification for the AMARANTH trial was a single ‘snapshot’ classification approach – using only the data from single time points, rather than modelling the timeseries of clinical measurements. Developing such models would be extremely useful for understanding neurodegenerative conditions (e.g [24]), capturing and predicting disease stages (beyond Aβ and tau deposition [7]), identifying key biomarkers per stage, and would inform both the duration of clinical trials and the timing of interventions.

Finally, in reusing data from a historical trial, our assessment of the use of AI in clinical trials is, by definition, post-hoc. The ML models had not been created when the study was originally designed, so their use did not form part of the statistical analysis plan. Future prospective validation of AI tools (e.g., formally locking the subgroup definition before trial initiation) will be important to build confidence for their widespread adoption. Nevertheless, it is important to note that the ML model’s parameters were blind to the trial data (i.e., complete independence of data sets) and that the model was given no information about the outcome of the trial (i.e., only baseline data before treatment). Next, we discuss how the findings can be instructive in the design of future clinical trials.

### 2.1 Modelling trial sensitivity

From the reanalysis of the AMARANTH trial, it is clear that identifying patient subgroups has the potential to increase the sensitivity of a trial to reveal a therapeutic effect that would otherwise be masked by differences between the subtype or stage of patients within a larger sample. To illustrate a generalised model for this process, consider the simulations presented in Fig. 2c. We model the change in cognition over a two-year period by distributions (probability density functions) for three population subgroups. ‘Stable’ patients (green) have little change in cognitive function, while ‘Slow’ (yellow) and ‘Rapid’ (purple) that involve increasing levels of cognitive decline, where between-patient variability increases (i.e. wider spread) as cognitive decline increases. The level of decline across the whole population (i.e., the mix of different subtypes) is shown by the solid blue curve – that is, the envelope of the whole population.

Consider what happens under administration of a drug that is only effective in reducing decline for the ‘Slow’ patient subtype (bottom portion of the figure). While the difference in the distributions of decline for patients on vs. off the drug is clear for ‘Slow’ patients (contrast the position of yellow distributions between top and bottom), at the whole population level (blue curves) it is much less obvious. We quantify using the Area Under the Curve (AUC) in a Receiver Operating Characteristic (ROC) analysis, a standard method for a diagnostic test (Fig. 2c). If a trial includes only ‘Slow’ patients, the treatment produces clear difference between the measured samples (AUC=0.87) while this is much harder to detect for a population with equal proportions of ‘Stable’, ‘Slow’ and ‘Rapid’ patients (AUC=0.56). In a clinical trial sample, the mix of underlying subpopulations is typically unknown – introducing random variability into the trial and making it harder to detect a true positive. In the case of the AMARANTH trial, the patient selection process resulted in a trial sample that subsequent analysis revealed was composed of approximately one third of ‘slowly progressive’ patients, two thirds ‘rapidly progressive’ and only a handful of ‘stable’ patients.

It is important to understand that the choice of patients with slowly progressive dementia is illustrative. Lanabecestat has a therapeutic action related to β-amyloid, making it suitable for patients in earlier stages of AD. A therapeutic targeting tauopathy stages may be more suited to rapidly progressive patients. Moreover, the labels ‘stable’, ‘slow’ and ‘rapid’ themselves are categorical descriptors derived by learning from disease trajectories in patient cohorts used for model training. Underpinning the labels is a continuous prognostic score that we derived from a multimodal AI model and binned into different groups based on normative data (considering data over 3-years). However, like any metric, there is inherent uncertainty in the measurements, and a small difference in prognostic score can lead to a change in a patient’s categorisation. Scores therefore represent an estimate of the patient’s state, subject to uncertainty in the measurements and the performance of the ML model. This uncertainty can be estimated using statistical techniques [25] and potentially reduced as biomarker precision improves to the point at which AI enables the integration of signals to precisely pinpoint an individual to within the stage-by-subtype space (Fig. 1c).

### 2.2 AI generalizability

Understanding how well results from a clinical trial generalize to new settings and populations is critical for any study. The clinical and scientific communities have accumulated knowledge to evaluate how well a particular study sample (e.g., a group of patients recruited from medical centres in North America and Europe) models broader populations (e.g., patients from around the globe) by considering a range of biological and demographic factors. While far from perfect, such human intuitions into which differences ‘matter’ for generalization underpin regulatory frameworks and commercial contracting.

Introducing AI tools into patient for selection can complicate understanding of how well a study result will generalize, particularly if the AI model is an 'opaque (black) box' model whose rationale for selecting specific individuals is not transparent. In particular, an AI model could learn a reliable association between multiple data features and a particular clinical presentation that works well for a specific set of training and test data. However, if we cannot map the AI engine’s operation to our intuitive understanding of the features used by the model it will be hard to evaluate how well the model will generalize to new data sets, in different contexts, geographies and for patients with different demographic backgrounds.

When developing the ML engine used for reanalysis of the ANMARANTH trial, we used two principal ways to address the challenge of AI generalizability. First, we adopted a metric learning approach that is “interpretable-by-design”, meaning that the model’s decision-making process (i.e., the features used by the model and their weights) can be traced and understood transparently, so the operation of the AI tool can be fully understood. Second, we tested generalisation performance by evaluating a family of trained ML engines on different data sets, demonstrating reliable stratification performance for data obtained in different research studies, as well as real-world memory clinics, from North America, Europe and Asia [[21], [22], [23]]. This provides reassurance that using this ML engine for clinical stratification is robust to different populations and contexts, while further work is ongoing to evaluate this family of models on data from non-AD dementias and underrepresented patient groups.

## 3 Improving clinical trial efficiency

**Fig. 3:**
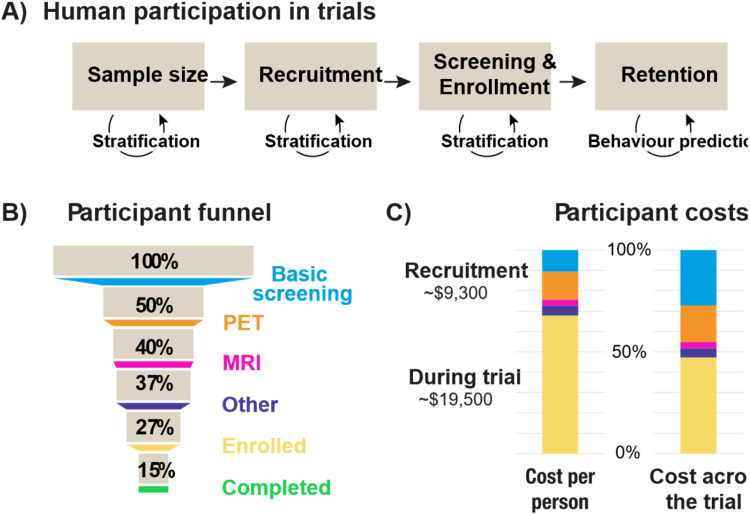
A) Overview on human participation in clinical trials and how AI can help. B) Patient recruitment screening flow modelled on two AD trials [32]. Of 100 people considered for a trial, only 15 on average complete the full protocol. C) Estimated costs related to procedures in the trial (based on US estimates). Basic screening and cognitive testing estimated at $3056, PET $4000, MRI $850, Other (e.g. genetics, blood) $1400. Costs during the trial encompass multiple MRIs, PET, cognitive tests, bloods / CSF, participant compensation and travel.

We have seen how AI-guided patient selection can increase the sensitivity with which therapeutic efficacy is measured. This has direct implications for the efficiency of clinical trials: if we have a more sensitive measure, we need fewer patients to assess the therapeutic to a standard statistical significance threshold (e.g., α <0.05). In this section, we consider how AI methods can be used to optimise human participation in clinical trials (Fig. 3a).

We start with trial design considerations related to sample size. We conducted statistical power analyses on the results from the AMARANTH trial with- and without- AI-guided patient stratification [20]. The results (Table 1) show dramatic reductions in the number of patients that need to be included within a trial for 90 % statistical power to a chosen level of statistical significance.

**Table 1:** Power analyses based on results from the AMARANTH trial showing the number of participants required to test lanabecestat 50 mg vs. placebo with 90 % statistical power (1-β) at different thresholds for statistical significance (α). The table contrasts the original sampling technique to an AI-stratified approach, producing sample sizes around 90 % smaller.

	α = 0.05	α = 0.01	α = 0.001
Original	1524	2396	3520
Stratified	164	234	328
Reduction	89 %	90 %	91 %

Reducing the sample size has obvious potential to reduce the costs of a trial. The per patient costs of clinical trials for drug development in the central nervous system are around $40,000 [26]. Depending on trial design, using stratified patient samples translates into potential cost savings of $54–$126 million per trial (Table 1), underscoring the financial impact of AI-guided stratification. Higher statistical power provides the opportunity for more agile trial designs. For instance, testing a broader range of dosing regimen or using adaptive trial designs [27,28] that enable optimization of trial parameters based on interim results.

Repowering a trial in this way requires a high degree of confidence that a particular group of patients has the right profile for a specific therapeutic. This may be justified by earlier phases of clinical development, but, in many circumstances, there may be uncertainty about which subgroups would benefit most from the treatment. In these cases, a trial could be designed to sample different subgroups in a pre-planned way. For instance, using stratified randomisation [29] to ensure that there is a balance of subgroup types across the different experimental conditions, with a pre-specified definition of the statistical analysis plan for subgroups. Adaptive enrichment might also be deployed, using an interim analysis (overseen by an independent data monitoring committee) to enrich the sample for the group that appears to benefit most from the treatment (e.g., response-adaptive randomization) and/or stopping enrolment for non-responding subgroup(s) (e.g. enrichment using futility rules) [30,31]. Finally, multimodal markers derived from AI-guided tools could provide interim trial endpoints that may be more sensitive than single markers for identifying treatment responders.

Implementing patient stratification could encompass the entire recruitment funnel from initial screening to the point of enrolment. We analysed recruitment across major trials of AD therapeutics [12,14,[17], [18], [19],^32^] involving around 50,000 patients. This showed a screen out rate of 72.9 % - i.e., for every 100 people screened, only 27 of them are enrolled into an Alzheimer’s trial.

Fig. 3b breaks down different screening stages in the clinical trial process for AD, modelled on the EMERGE (NCT02484547) and ENGAGE (NCT02477800) trials [32]. In Fig. 3c we further estimate costs (using US prices) based on these trial designs in terms of the raw cost per participant, and the proportionate cost across the whole trial. It is apparent that while the largest cost at the single patient level is incurred during the trial, total expenditure is greater during the recruitment and screening phases. Indeed, more cost can be incurred on the three quarters of patients who do not make it into the trial than those who stay in the trial for two years. Trials would be more cost efficient if more of the patients entering the top of the funnel ended up being recruited to the trial – i.e., getting it right first time. How might AI help?

The stratification models we considered for the AMARANTH trial relied on MRI, PET and genetic information to support prediction and patient stratification. These specialist data are expensive and most suited to making a final decision on participant enrolment based on the highest possible precision in stratification. However, predictions can be derived from less-invasive, lower cost data (e.g. blood tests, cognitive tests, electronic healthcare records). While the features used by a model affect its accuracy [21], the ability to perform a first pass at the top of the funnel that is more accurate than standard screening methods has significant potential to enhance the lower portions of the enrolment funnel.

Implementing stratification at the point of recruitment (i.e., via ethically consented analysis of data contained within electronic health records [33]) and/or at the point of basic screening can reduce patient burden, lower costs, and speed up trial recruitment. In terms of trial efficiency, this will lower the proportion of resources expended on participants who are not enrolled in the study. Pre-screening using AI models to identify likely candidates from electronic health records (prior to formal screening), would increase the probability that a given patient is enrolled in the study before the individual has any active contact with the study and any new tests are run.

Recent advances in blood-based biomarkers are opening new opportunities to lower patient burden and refine inclusion within clinical trials [3]. The plasma-derived assay pTau217, for instance, provides a means of detecting elevated amyloid pathology using a much less costly or invasive measure than PET imaging. Current trial protocols envisage its use as a preliminary measure that is confirmed by PET imaging (e.g., [34]) and recent FDA approval [35] means it may be used without confirmatory PET. Further, recent advances in proteomics and large-scale datasets (e.g. GNPC [36]) hold great promise for the discovery of precision biomarkers and new ADRD subtypes. However, blood markers or proteomics provide single sources of information that should be statistically combined with other markers (e.g. MRI scans, genetics, neuroinflammation, cognition) to provide the best basis to pinpoint an individual’s disease subtype and stage (Fig. 1). AI-guided tools synthesising blood and proteomics markers can match patients with different pathology profiles and at different progression stages to the right targets. This has strong potential to facilitate the design of multi-arm trials that test multiple targets against the same placebo group, accelerating and enhancing the efficiency of clinical trials.

Beyond recruitment, trial efficiency is also affected by participant retention in trials. Unfortunately, not every participant that enrols into a clinical trial is able to complete it. A range of factors influence drop out including mortality and morbidity, adverse events, patient burden, mental health comorbidities and worsening of disease state [37,38]. While some of these factors are outside the control of study investigators, AI models designed to predict behaviour and identify risk of dropout have potential to be used for proactive monitoring and early intervention to facilitate continued engagement in a trial. This can be particularly important for underrepresented populations [39] where support for patients and caregivers can reduce drop out from trials. Finally, interpretable AI models allow us to determine key combinations of predictive markers for patient stratification, allowing smarter and more efficient selection of data types to be collected at different clinical trials stages, accelerating trials and enhancing retention rates.

## 4 Improving therapeutic effectiveness

Successfully executing a clinical trial to show the efficacy of a new therapeutic provides the foundation for regulatory approval. However, it does not guarantee real-world success or reimbursement. In this section we consider how AI approaches can support precision treatment for the best use of therapeutics, as well as key considerations in evaluating AI tools when used in wider populations.

### 4.1 Adoption in clinical practice

The challenge of accurate diagnoses in dementia has been a key contributing factor to the lack of success in therapeutic development over the past two decades [1]. Yet clinical trials represent a high resource environment that use costly biomarkers. What are the prospects for accurate diagnosis in real world healthcare that is typically less well-resourced than a clinical trial?

A range of AI methods have been explored to enhance dementia diagnosis, with a particular focus on AD and the interpretation of imaging data [40,41]. Such systems could be useful adjuncts to the interpretation of radiological data, but as outlined above (Fig. 1), any one diagnostic marker will give an incomplete picture of the patient’s disease state. Multimodal approaches [22] are critical, particularly when used for differential diagnosis to identify subtypes of dementia and their overlap [42].

How could multimodal models be useful when different types of data are available in different clinical settings? We can conceptualise the process as a “Russian Nesting Doll (Matryoshka)” family of models: constructed using the same architecture and functional goal, but using different input features that influence the precision and specificity of the predictions that are produced. We have seen that a model trained with PET, MRI and ApoE4 information can precisely separate patients in the context of a clinical trial [20]. We conceptualise this as the ‘core’ of the Russian Doll – providing us with the most tightly defined sense of where a patient sits in the space of dementia stage and subtype. Within a secondary care setting, typical data that a clinician has access to would be MRI, a cognitive test, and demographic information. This situates the patient, although less precisely than the core model. Finally, within primary care, models that integrate demographics and cognitive data would provide more information to the clinician than a memory test alone. This approach can benefit from using a ‘privileged information’ [43] framework where models are trained using richer data than are available at test time, allowing for robust predictions even within more limited inputs. For instance, training a model on MRI, cognitive data and demographics, and applying it in a setting where only cognitive data and demographics are available. Integrating developments in scalable, remotely-collected data through mobile technology (e.g., RADAR-AD consortium [44]) has the potential to enrich AI models and democratize diagnosis in community settings. While we have example instantiations of these models [21], further work is needed to provide full validation across a family of models and build tools that bridge drug discovery with adoption of therapeutics in healthcare.

Designing and implementing models that are compatible with existing clinical workflows is key for advancing dementia therapeutics. The ‘best’ model is not necessarily the one with highest performance, but rather the one that has most chance of improving clinical pathway decisions that can range from deciding that the patient needs an onward referral to making a refined choice between two specific medicines (i.e., its clinical utility). Ultimately, the AI models that produce most clinical impact may be those that are (a) most easily integrated within Electronic Healthcare Record systems and (b) robust to real world healthcare data that often contains missing or degraded data. Multimodal approaches are inherently more robust than single markers, and statistical approaches based on probabilistic imputation of missing data can further support real world deployment in healthcare [[45], [46], [47]].

Finally, it is worth considering the computing resource requirements associated with running AI models. Large AI models can necessitate large data flows and extensive computation. As AI usage becomes ubiquitous, these considerations may reduce, but currently low-bandwidth data networks and/or lack of dedicated IT hardware in clinical settings can introduce barriers to adoption. These barriers may be particularly acute in lower- and middle-income country settings [48,49].

### 4.2 Interpretability

Many AI algorithms operate as ‘opaque (black) boxes’ that make their operations difficult to understand. Deep neural networks can involve many millions of free parameters, making the operation of the system hard to understand. This lack of transparency is a challenge when the system may be informing clinical decision making and treatment planning [50]. Interpretability varies according to the types of machine learning used in a solution, and solutions can be made ‘interpretable by design’ [42,51]. Where this is not possible, techniques can be used to infer or visualise how the system is operating to make solutions more interpretable. For instance, reverse engineering features to test their clinical relevance (e.g., relationships between biomarkers and cognitive decline), and/or implementing methods based on the integration of concepts [52], attention [53] and logic [54]. As outlined above, interpretability considerations are related to the ability of humans to assess the likelihood of AI model generalizability.

### 4.3 Algorithmic bias

It is critical to understand the potential for AI models to entrench or even widen existing health inequalities. When AI models are trained on specific data sets, their parameters are tuned to the properties of those data. Clinical trials and research cohorts generally overrepresent majority populations [55,56] creating the potential for models to perform poorly when presented with data from underrepresented groups. This is particularly critical when building models for ADRD given known increased risks in specific racial/ethnic groups [57,58]. Further, when AI models have been trained using diagnostic labels derived by clinicians, there is potential for AI to amplify biases in human decision-making through the automated use of AI tools at scale. AI methods can be used to detect algorithmic bias (e.g [59]), however rigorous testing of generalization to minority populations, and improving the diversity of the underlying datasets used to train models are critical to longer term efforts. Real-world data sets (as opposed to research cohorts) are advantageous in this regard as they tend to be more broadly representative of the underlying population.

Finally, it is important to consider that models should not be static: clinical standards evolve, data types are refined, and models being applied in real world practice may becoming increasingly divergent from the populations on which they were trained. It is critical therefore to ensure continued model relevance. Interpretability can help with this, but specific procedures can be used to quantify drift (i.e., differences between training and test data sets) [60] so that models can be retrained or recalibrated once deployed. Addressing bias, maintaining model relevance, and improving dataset diversity are all critical to ensuring equitable access to AI-enhanced dementia care.

### 4.4 Regulatory considerations

As AI tools move from research into clinical practice, regulatory oversight becomes essential. Adopting AI stratification within healthcare is likely to require regulatory approval as a diagnostic technology – either as a companion to a new medicine or as a diagnostic algorithm that would support clinicians in treatment planning. Regulators around the globe are currently grappling with the right way to balance the risks and opportunities of AI for patient benefit. Much of this involves extending thinking about patient safety, the efficacy of solutions, risk stratification, and software lifecycle management that has been part of Software as a Medical Device (SaMD) for two decades (e.g., FDA guidance published in 2005). Specific additional elements relate to the adoption of Good Machine Learning Practice principles outlined jointly between US, UK and Canadian regulators [61]. These are designed to ensure that models are robust, well validated, secure, transparent and that performance is actively monitored once products are introduced into the market (i.e., post-market surveillance). Where AI algorithms can update themselves, or produce highly variable outputs (e.g., natural language), regulators require performance monitoring plans to ensure ongoing safety and effectiveness as AI models evolve under real-world usage.

In considering the Regulatory approach, there are unresolved questions about the relationships between AI tools used as part of a clinical trial, and those required once a new medicine has been approved as safe and effective. In particular, if the success of the trial depends in part on the use of an AI-guided stratification tool, does the tool become essential for prescribing the therapeutic in healthcare settings? Here, we see significant potential for “Russian doll” families of models that are related, but use lower-cost less-invasive types of data from those typically collected within a clinical trial (e.g. blood tests and cognition instead of PET scans). This could result in the use of simple, interpretable models that aid clinicians to assign patients to the right treatment and can be easily related to quantities already known and trusted by Regulators and clinicians.

Ultimately Regulatory decisions will be guided by evidence that the use of a particular tool is safe and effective. Because clinical trials already collect multimodal data, including lower cost data, there is potential to simultaneously validate the use of ‘gold standard’ stratification tools alongside tools that have practical use in real world clinical settings. This will necessitate comparing patient inclusion/exclusion criteria across different models, and relating these to outcomes in the placebo and treatment groups. Similar considerations are needed for thinking about AI-guided patient selection at different stages of the recruitment funnel, particularly to ensure that patients with particular demographics are not being systematically excluded from trials.

## 5 Conclusion

The search for effective treatments for Alzheimer’s and related dementias has long been hindered by patient heterogeneity and the lack of sensitive tools for stratification. In this review, we have explored how advances in AI—particularly those leveraging multimodal data—can enhance both the development and deployment of new therapeutics.

Multimodal approaches enable more precise identification of a patient’s disease subtype and stage, matching patients based on their pathology profile and stage to targets. This has strong potential to transform clinical trial outcomes by increasing statistical power, improve operational efficiency through adaptive design of multi-arm trials, and pave the way to precision medicine and combination therapies in ADRD. These benefits extend beyond clinical trial settings, opening up pathways to more targeted healthcare in real-world settings.

However, realizing the full potential of AI in dementia care requires that we address key challenges: ensuring model transparency, fairness, and generalizability across diverse populations of patients requiring dementia care, and making tools compatible with clinical workflows and resource constraints.

AI alone will not solve the complex challenge of ADRD. But, when integrated with scientific insight, clinical expertise, and lived experience of patients and caregivers, intelligent analytics can accelerate the discovery and delivery of diagnostics and therapeutics—ultimately transforming dementia care and improving outcomes for individuals and their families worldwide.

## Declaration of competing interest

The authors declare the following financial interests/personal relationships which may be considered as potential competing interests: Zoe Kourtzi, Andrew Welchman report a relationship with Prodromic that includes: board membership.

## Glossary of Terms

Clinical Label: Diagnostic category assigned to a patient (e.g., MCI, AD).
Machine Learning (ML): Algorithms that learn patterns from data to make predictions/decisions.
Metric Learning: ML approach that separates similar patients that are spatially close to each other by learning discriminable prototypes.
Probability Density Function (PDF): A curve describing how likely different outcome values are within a population.
SaMD (Software as a Medical Device): Software intended for medical purposes.
Supervised Learning: ML algorithms trained on labeled data.
Un- /Semi‑Supervised Learning: ML algorithms that learns from unlabeled or a mix of labeled and unlabeled data.
